# Accuracy and trending abilities of finger plethysmographic blood pressure and cardiac output compared to invasive measurements during caesarean delivery in healthy women: an observational study

**DOI:** 10.1186/s12871-020-01078-8

**Published:** 2020-06-27

**Authors:** Ivar N. Omenås, Christian Tronstad, Leiv Arne Rosseland

**Affiliations:** 1 Division of Emergencies and Critical Care, Oslo University Hospital, Oslo, Norway; 2 Department of Clinical and Biomedical Engineering, Oslo University Hospital, Oslo, Norway; 3 Institute of Clinical Medicine, University of Oslo, Oslo, Norway

**Keywords:** Blood pressure, Cardiac output, Caesarean section, Validation studies, Bland Altman, Polar plot

## Abstract

**Background:**

In women presenting for caesarean section under spinal anesthesia, continuous measurement of circulatory aspects, such as blood pressure and cardiac output, is often needed. At present, invasive techniques are used almost exclusively. Reliable non-invasive monitoring would be welcome, as it could be safer, less uncomfortable, and quick and easy to apply. We aimed to evaluate whether a non-invasive, finger plethysmographic device, the ccNexFin monitor, can replace invasively measured blood pressure in the radial artery, and whether cardiac output measurements from this device can be used interchangeably with measurements from the mini-invasive LiDCO monitor currently in use at our institution.

**Methods:**

Simultaneous invasive measurements were compared to ccNexFin in 23 healthy women during elective caesarean section under spinal anesthesia. We used Bland Altman statistics to assess agreement, and polar plot methodology to judge trending abilities with pre-defined limits.

**Results:**

Mean arterial and systolic pressures showed biases (invasive – ccNexFin) of − 4.3 and 12.2 mmHg, with limits of agreement of − 15.9 – 7.4 and − 11.1 – 35.6, respectively. The ccNexFin trending abilities were within the suggested limits for mean pressure but insufficient for systolic pressure compared to invasive measurements. Cardiac output had a small bias of 0.2 L/min, but wide limits of agreement of − 2.6 – 3.0. The ccNexFin trending abilities compared to the invasive estimated values (LiDCO) were unsatisfactory.

**Conclusions:**

We consider the ccNexFin monitor to have sufficient accuracy in measuring mean arterial pressure. The limits of agreement for systolic measurements were wider, and the trending ability compared to invasive measurements was outside the recommended limit. The ccNexFin is not reliable for cardiac output measurements or trend in pregnant women for caesarean delivery under spinal anesthesia.

**Trial registration:**

Registered May 23, 2013, at ClinicalTrials.gov under number NCT01861132.

## Background

In a normal pregnancy, large alterations occur in the circulatory physiology. Blood volume, stroke volume, heart rate, and cardiac output (CO) increase, where blood pressure (BP) and peripheral resistance decrease [1]. During caesarean section (CS), both spinal anesthesia and oxytocin administration can precipitate severe drops in peripheral arterial resistance and BP. Vasopressors and inotropes are administered to counteract the changes in BP and CO [2]. Perioperatively, BP is usually measured intermittently using oscillatory devices, and changes of short duration are mostly unnoticed, even if substantial. In healthy parturients, changes in BP and CO are usually well tolerated and easily corrected, but in patients with pre-eclampsia or cardiac disease, close monitoring of hemodynamic variables is necessary to prevent harm to the mother or fetus. Cardiac disease often goes undiagnosed in pregnant women; of maternal deaths from a cardiac cause in the UK and Ireland in 2009–2014, 77% did not have a known pre-existing cardiac condition [3].

Anesthetists are trained to observe and interpret heartbeat-to-heartbeat variables and often need to see trends and changes over time. In our department, the current standard tool for continuous cardiovascular monitoring is the LiDCOplus monitor (LiDCO Ltd., London, UK). LiDCO offers continuous data on BP, peripheral resistance, and CO, among other variables. However, the monitor requires intra-arterial access and is reserved for delivery in high-risk pregnancies.

It would be of great advantage for research and clinical monitoring of pregnant women if detailed and reliable continuous measurements could be obtained using non-invasive technologies. Advanced monitoring could be established more quickly and easily, and with less risk. For example, the ccNexFin monitor (NexFin Systems, BMEYE, Amsterdan, NL) is non-invasive and easy to apply. There is, however, currently insufficient evidence that non-invasive monitors are reliable for pregnant women.

The objectives of the present study were to assess the agreement and trending abilities for systolic and mean arterial BP between ccNexFin and invasive BP measures, assess the agreement and trending abilities for CO between the ccNexFin and LiDCO monitors, and determine if ccNexFin can replace invasive measurements of BP and CO during CS under spinal anesthesia in healthy, pregnant women. The expected outcome was that the non-invasive measurements and trending abilities of BP are reliable compared to invasive measurements, but we expected CO to be less reliable.

## Methods

### Ethics approval

The study was approved by the Regional Committee for Medical and Health Research Ethics, Southern Norway (REC ID: 2012/1155 approved 01/02/2013). All participants provided written informed consent. The study followed the Declaration of Helsinki and was conducted according to Good Clinical Practice.

### Patient population

Healthy, non-smoking, normotensive women with singleton pregnancies scheduled for elective caesarean delivery under spinal anesthesia were asked to participate in this study. Participants were recruited in collaboration with “The Placenta Project” (REC ID: 2011/2419) and, for this subset of the study population, a common information leaflet was developed. The information was released during the recruitment period and included detailed information about the anticipated pain during arterial cannulation and the extra time needed when establishing both invasive and non-invasive monitoring. As part of the study protocol for “The Placenta Project”, all participants received an intra-arterial line used for blood sampling at delivery. The protocol for The Placenta Project and description of the entire recruitment period was published previously [4]. Exclusion criteria were considerable pre-existing morbidity, pregnancy complications, contractions prior to scheduled C-section, and prior Raynaud phenomena, as this is not compatible with use of the ccNexFin monitor. Two women with hypothyroidism, each supplemented with a low dose of L-thyroxine (50 and 75 μg daily) and one woman with mild asthma and occasional use of salbutamol (not taken in the days prior to participation in the study) were included in the study. The inclusion period was from May 2013 to January 2014. Demographics of the study population are given in Table 1.

**Table 1:** Patient demographics

	Mean (SD)	Range
Age, yr.mo	35.10 (3.2)	29.6–42.7
Height, cm	167 (5.0)	160–180
Weight before pregnancy, kg	64.0 (10.7)	50–91
Weight at delivery, kg	78.9 (12.3)	60–105
Length of pregnancy, days	275 (6.5)	260–292

### Monitoring devices

The LiDCOplus monitor (LiDCO Ltd., London, UK, version 4.02.95) is in routine clinical use and has documented accuracy and trending abilities [5]. This monitor provides information about circulatory changes from heartbeat to heartbeat and is used when advanced monitoring is indicated, such as during major surgery, or during interventions on patients with circulatory disorders. Mathematical analysis of the intra-arterial pressure curve is performed with pulse power analysis using the built-in software PulseCO. The LiDCO monitor estimates many aspects of the circulation and can be used with or without lithium dilution calibration. In this study, we used calibrated CO, aiming for optimal accuracy. This technology has been used in studies of healthy women and pregnant women with heart disease [6], and is the standard method for perioperative monitoring of pre-eclamptic women at our institution. In the comparison of CO methods, LiDCO serves as the reference.

The ccNexfin monitor (NexFin Systems, BMEYE, Amsterdan, NL, version 1.9.0.1001) is based on the principle of the unloaded vascular wall [7], the Physiocal criteria [8], and a generalized waveform filter to reconstruct brachial pressure from finger pressure [9]. An inflatable cuff is placed around one of the three middle fingers of either hand. An integrated plethysmograph measures the volume of blood under the cuff using an infrared light source and a photosensor. The monitor initially determines a set point for the finger cuff pressure at which most of the venous blood is displaced and the arterial diameter is reduced to no more than 50% of the expanded diameter. The set point is intermittently calibrated according to the Physiocal criteria to account for changes in the vascular state of the finger. The cuff pressure is continuously adjusted to counter the varying intra-arterial BP, keeping the signal from the photosensor and, consequently, the blood volume and arterial diameter under the cuff constant. This way, the artery wall is said to be unloaded, transmural pressure is zero, and the pressure in the cuff represents the intra-arterial pressure. The measured finger BP is transformed to reflect brachial BP. The CO calculations in the ccNexFin are based on pulse contour analysis of the derived arterial pressure curve. The monitor is designed to work without external calibration. Our research group has been involved in several projects using finger plethysmographic monitor technology, including the largest population-based study ever utilizing this technology, The Tromsø Study [10].

### Study design

In this prospective observational study, a 20G BD arterial cannula (Becton Dickinson Infusion Therapy Systems*,* Inc*.*, Utah, USA) was placed in the radial artery after skin infiltration with lidocaine (5–10 mg). The cannula was connected to a Siemens Dräger Infinity Gamma XL hemodynamic monitor (Drägerwerk AG & Co. KgaA, Lübeck, Germany) via a Codan X-trans pressure transducer (CODAN pvb Critical Care GmbH, Forstinning, Germany) and the signal calibrated according to standard departmental procedures. Peripheral IV catheters were placed on both arms.

Intra-arterial BP data was passed through to the LiDCOplus monitor. Invasive heart rate (HR_inv_), invasive systolic arterial pressure (SAP_inv_), and invasive mean arterial pressure (MAP_inv_) were recorded at a rate of one sample per heartbeat. The CO_LiDCO_ estimated by PulseCO was also recorded. A single point calibration of CO was performed. The ccNexFin monitor was applied to one of the three middle fingers on the same arm as the intra-arterial cannula and corresponding variables (HR_nex_, SAP_nex_, MAP_nex_, and CO_nex_) recorded.

While sitting on the operating table, the subjects received spinal anesthesia with bupivacaine (10 mg) and fentanyl (20 μg) using a 27G pencil point needle. Co-loading with intravenous 0.9% NaCl (1000 mL) was started. The parturients were then placed in the supine position with a left lateral tilt using a wedge under the right hip. Immediately after injection of the drugs, an intravenous bolus of phenylephrine (25–50 μg) was given, followed by an infusion starting at 0.25 μg/kg/min and titrated according to invasive BP, aiming for a stable SAP_inv_ > 90 mmHg.

### Data recording

To acquire synchronous sampling from both monitors, measurements were sampled in real time by the same computer. Samples were acquired through the RS232 port of the LiDCO monitor and the analog output from the ccNexFin monitor using a data acquisition card and software from National Instruments. This setup was evaluated for electrical safety and approved by the appointed committee at Oslo University Hospital.

Time-stamped data for inter-beat interval (IBI), SAP, MAP, and CO from both monitoring devices was recorded to a single dataset per subject, one sample per heartbeat, using software developed in-house using National Instruments LabVIEW®. Events were marked in real time and saved to a file using the same software.

Due to subject movement following spinal anesthesia, placement of a hip wedge, and adjustment of the arterial pressure transducer and the ccNexFin heart reference system, we considered data from the first 2 min after spinal anesthesia as unreliable. The arterial line was used for blood sampling just prior to delivery, causing a pause in our registration, and following delivery there was again more subject movement causing unreliable data. It is also the experience of our group that the LiDCO needs recalibration after delivery [11]. We included the data between 2 and 12 min after spinal anesthesia for our calculations.

### Statistical analysis

Due to differences in processing time between the two monitors, the LiDCO samples were ahead of the ccNexFin samples, by approximately two heartbeats on average, though they were sampled synchronously from the outputs of the monitors. For each session, this difference was adjusted by calculating the lag using a cross-covariance analysis of the IBI time-series, which are assumed to be equal between monitors, and then shifting the ccNexFin recording ahead by the calculated lag in order to align the recordings for comparison at equivalent beats. This was performed in Matlab R2014b (Mathworks, Nantick, Massachusetts, USA).

Artefacts were reduced using a previously published method for detecting and removing outliers in continuous BP and CO recordings [12]. Data points for statistical analysis were constructed with 1-min intervals by averaging data over the first 10 s of each minute.

Methods were compared using Matlab and Stata v15 (Statacorp LLC, College Station, Texas, USA). We used the method first described by Bland and Altman to investigate the agreement of the ccNexFin monitor with invasive BP and LiDCO CO measurements [13, 14]. Early versions of this method did not sufficiently consider the structure of the data and could produce too narrow limits of agreement, and too narrow confidence intervals, with repeated measurements per subject. We calculated limits of agreement based on the repeated observations method as described by Zou [15]. Confidence intervals for the limits of agreement were calculated using the MOVER algorithm [15]. This method is preferred when the true value varies, when there is a different number of measurements from each subject, and when the between-subject variance is large with respect to the within-subject variance. The MOVER method also allows the construction of asymmetric CIs. Diagnostic plots suggested by Bland and Altman were inspected to check for underlying assumptions. The high number of repeated measurements increases the information and the statistical power. However, sample size was decided without a formal power calculation.

Polar plots were used to assess trending abilities for both BP and CO [16]. As suggested by Critchley [16], the smallest changes were considered to most likely represent noise and were excluded from the Polar plot analysis. Data points with an average change from the previous measurement of more than 5 mmHg for BP or 0.5 L/min for CO were included.

## Results

**Fig. 1:**
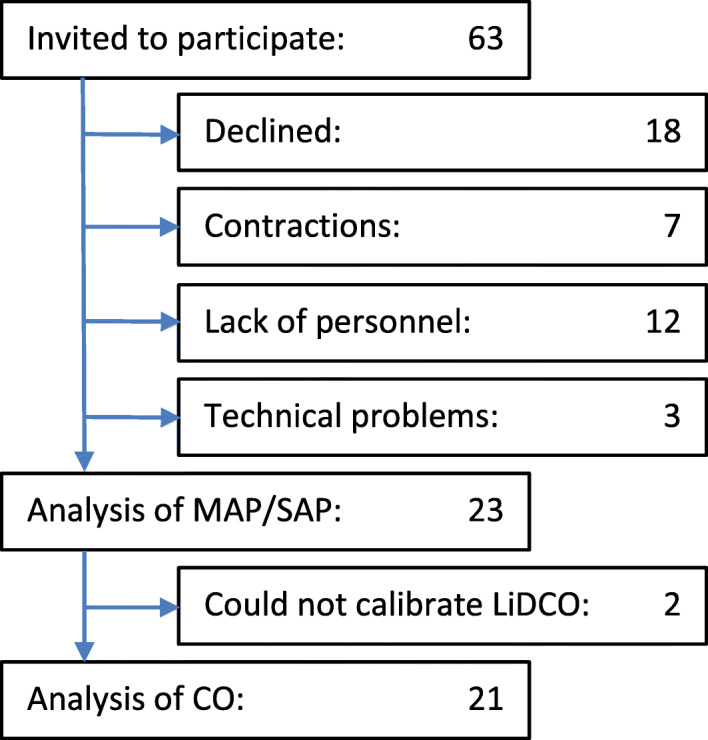
Flow chart of patient screening, inclusion, and analysis

Of 63 subjects approached to participate in the study, 45 agreed to participate. Of these women, 7 went into labor prior to the CS, and study personnel were not available at the time of the CS for 12. Another two women were excluded due to technical problems with the recording equipment, and in one woman we did not succeed in placing an intra-arterial line. This left us with 23 participants eligible for BP analysis. Two additional women were excluded from the analysis of CO due to unsuccessful calibration of the LiDCO monitor (Fig. 1).

Mean (SD) time from spinal anesthesia to blood sampling was 16.1 (5.5) minutes, with values ranging from 5.6 and 31.7 min. We had some missing data, mostly due to delivery sooner than 12 min after spinal anesthesia, or misalignment of the ccNexFin heart reference system. For the BP analysis, 209 of a theoretical maximum of 230 data points remained (90.9%). For the 21 participants included in the CO analysis, 187 of 210 possible data points remained (89.0%).

**Fig. 2:**
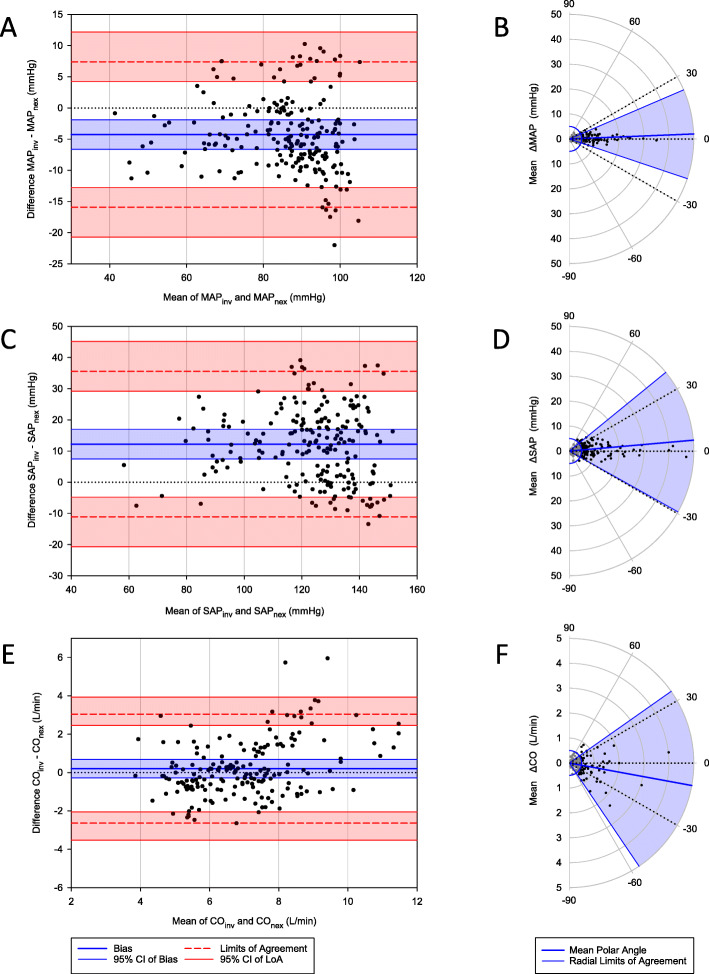
Comparison of invasive measurements with ccNexFin. **a** Mean arterial pressure (MAP) in a Bland Altman plot and **b** Polar plot. **c** Systolic arterial pressure (SAP) in a Bland Altman plot and **d** Polar plot. **e** Cardiac output (CO) in a Bland Altman plot and **f** Polar plot

The Bland Altman plots and Polar plots are shown in Fig. 2, and the limits of agreement and Polar plot analyses are shown in Table 2.

**Table 2:** Bias (invasive – ccNexFin), limits of agreement (LoA), and Polar plot analyses

	Bias (95% CI)	LoA (95% CI)	Mean polar angle (SD)	Radial LoA
MAP	−4.3 mmHg (−6.6, −1,9)	−15.9 (−20.7, − 12.8) to 7.4 (4.2, 12.2)	2.3° (10.0°)	−18.6°, 23.2°
SAP	12.2 mmHg (7.5, 17.0)	− 11.1 (− 20.7, − 4.8) to 35.6 (29.2, 45.2)	5.0° (17.3°)	−29.2°, 39.2°
CO	0.2 L/min (− 0.3, 0.7)	− 2.6 (− 3.5, − 2.0) to 3.0 (2.5, 3.9)	−10.4° (27.2°)	−55.9°, 35.1°

## Discussion

Even though the anesthetist aims to keep the patient as stable as possible during caesarean delivery, rapid and major fluctuations in BP and CO are common. Both spinal anesthesia and injections of oxytocin result in sudden drops in the peripheral resistance, with decreases in BP and concomitant increases in CO [2, 17]. Standard clinical practice is to administer fluids and vasopressors [18]. Surgery, bleeding, anxiety, and discomfort also affect the cardiovascular system. Thus, this clinical setting is challenging for any monitoring device, especially regarding trending abilities. Could inclusion of parturients with pre-eclampsia or cardiac diseases have increased the hemodynamic challenge and added value to the evaluation? Pre-eclamptic patients tolerate spinal anesthesia with less of a change in BP and CO due to peripheral vasoconstriction [19]. Parturients with cardiac disease are another group in which invasive monitoring is indicated and part of standard care in our hospital. The patients usually receive spinal anesthesia, and vasodilation has to be carefully managed throughout the surgery. The hemodynamic response varies a lot depending on cardiac diagnosis and function [6]. Collecting a large sample of patients with invasive monitoring in obstetric anesthesia is challenging. Spinal anesthesia-induced vasodilation and hypotension is common and has a major impact in healthy parturients, and beat-to-beat monitoring is recommended to titrate prophylaxis and the treatment of severe hypotension. Implementing this into practice is important for clinicians who manage parturients with severe cardiac co-morbidity.

### Blood pressure varies by measurement site and modality

The definition of a gold standard for BP is debatable. Although auscultatory BP, measured on the upper arm, may still be considered the gold standard, it is too slow and impractical for use in the setting of caesarean delivery. For most patients, intermittent oscillometric measurements are sufficient, but in more challenging cases, continuous measurements are needed. These measurements are usually obtained invasively. For invasive measurements, the gold standard may be the BP in the ascending aorta, but this measurement is not obtainable. The radial artery is the preferred site, as it is conveniently located, easy to cannulate, and the incidence of complications is low [20]. Still, peripherally measured intra-arterial BP is not equal to the central pressure. The pressure curve is modified as it travels along the branches of the arterial tree by both the elastic properties of the central vessels and reflected waves from the periphery.

Oscillometric non-invasive BP measured in the upper arm underestimates high BP and overestimates low BP, whereas mean pressures are similar compared to intra-arterial measurements in the radial artery [21]. In 1990, Gravlee et al. [22], compared intra-arterial BP in the brachial artery measured with four methods of non-invasive BP measurement on the upper arm before, during, and after cardiopulmonary bypass. Averaging overall measurements, they found that the auscultatory method reported lower systolic, but higher mean and diastolic pressures than the invasive measurements, whereas the oscillometric method reported an equal systolic and higher mean and diastolic values compared to invasive measurements. Judging from the graphs, the same is true when considering the first two measurements (i.e. before open chest surgery) separately [22].

In 1951, Wood et al. simultaneously measured the BP in radial and brachial arteries in 17 healthy subjects and found that the SAP in the radial artery was 6 mmHg higher, and MAP and DAP slightly lower (2 and 1 mmHg), than in the brachial artery. For hypertensive subjects, the difference in systolic pressure was increased [23]. Pauca et al. measured pressures in the radial artery and in the ascending aorta during bypass surgery. They found small differences in MAP and DAP, but greater differences between centrally and peripherally measured SAP, with pressures in the radial artery being 12 mmHg higher on average. Systolic values were also found to have much greater variance than the mean and diastolic values [24].

The ccNexFin measures BP in a finger. The waveform is transformed to approximate invasive brachial BP [9] using a model that relies on measurements and assumptions in a group of 53 men, some healthy, and some with varying degrees of hypertension and cardiovascular disease. We found similar MAP values between the monitors, with ccNexFin reporting values an average 4 mm higher than the invasive measurements. Systolic values were approximately 12 mm lower on average. A slightly lower systolic value is expected, as ccNexFin aims to represent a more central pressure, but the difference is larger than can reasonably be explained by this effect. The inaccuracy is small and will likely be of minor importance during caesarean delivery under spinal anesthesia.

### Varying definitions of hypotension

An analysis by Klöhr et al. reviewing definitions of hypotension after spinal anesthesia for caesarean delivery found that, in research, relative limits seem to be more popular than absolute thresholds [25]. This and other papers reference a survey by Burns et al. from 2001 as an argument that anesthetists prefer absolute thresholds in this setting [26], but they actually did not. The authors claimed that a relative rather than an absolute decrease may be more important. They added that, not only the degree of hypotension, but also the duration may be important. Nevertheless, as absolute limits do not require a baseline to be determined, it may be reasonable to assume that this simpler approach would be preferred by many. In a recent consensus document, Kinsella et al. suggested taking a baseline BP measurement before spinal anesthesia using repeated measurements if the BP is not stable, if it is higher than expected, or if the woman is in labor [27]. They recommended aiming for a SAP ≥90% of baseline and to avoid a decrease to < 80% of baseline. The SAP was also suggested to be a less important variable than MAP as a determinant of organ perfusion. Recommended limits are still based on the SAP, as this has been the primary outcome in most of the available research. MAP is unlikely to be used to define hypotension in this clinical setting without more supportive data [27].

Auscultatory, oscillometric, invasive, and finger plethysmographic techniques use different principles to measure or estimate BP. In addition, measurements are made at different anatomic sites. It is important to be aware of the measurement techniques used in scientific studies, their definitions of safe limits for BP, and to consider the characteristics of these methods compared to the ones being used in clinical practice.

From this perspective, the inaccuracy of ccNexFin compared to invasive arterial pressure has a minor impact, and we recommend finger plethysmographic measurement for clinical use and in research.

### Trending abilities for blood pressure

The degree to which two monitors agree in their ability to track changes is also crucial. For the Polar plot method we applied, Critchley et al. suggest using a limit of ±5° for angular bias and radial limits of no more than ±30° for good trending abilities [16]. Only the MAP measurements satisfied these limits. The low angular bias suggests that the monitors were in good calibration, and with radial limits well within ±30° the ccNexFin exhibits good trending ability compared to intra-arterial measurements. The SAP had an angular bias on the border of the suggested range, but the radial limits were too wide. Taking into consideration the hemodynamic variations typical for caesarean delivery under spinal anesthesia, this is a challenging model, with larger intra-individual variations than in critical care patients. We recommend finger plethysmographic measurements in clinical settings and for research purposes requiring good BP trending ability, such as repeated measurements. In pre-eclampsia or other settings with pregnancy-induced hypertension, invasive measurements should replace the finger plethysmographic method due to the tendency to underestimate SAP.

### Cardiac output

Regarding CO, we did not find sufficient agreement between the monitors. Even though the bias was small, the limits of agreement were wide, more than ±40% of the mean CO. Both the angular bias and radial limits of agreement were far outside recommended limits, suggesting that ccNexFin cannot reliably track changes in CO. This is consistent with the results in a study comparing this technology to echocardiographic estimates of CO [28]. Based on the interpretation of the results, we do not recommend using ccNexFin to measure CO or to monitor CO trends in the clinic or research in pregnant women.

### Limitations

In this study, we presented the accuracy (bias) and precision (variability) of agreement between two methods. We calculated limits of agreement and assessed the use of ccNexfin against invasive measurements for BP and the LiDCO monitor for CO. As described by Hapfelmaier et al. [29], the precision of agreement partly depends on the precision of measurement (repeatability) of both devices. For example, the limits of agreement will become wider as a consequence of using an imprecise reference technique. This means that the agreement between a new technique and a reference technique needs to be judged in light of the precision that the techniques themselves are able to achieve [29]. In the present study, it was not possible to determine the repeatability (variation around a true value). Obstetric anesthesia is characterized by constantly changing hemodynamics, and repeated measurements during one constant value of BP or CO within the experiment is impossible. Thus, determining the precision of measurement of the LiDCO per se and the Nexfin technique per se was not possible.

A total of 21 patients were analyzed and a larger sample could have increased the precision and generalizability. However, it is important to take into consideration the large number of repeated measurements per patient and that the analyses included all of these data points to assess trending abilities.

## Conclusion

We consider the ccNexFin monitor to have sufficient accuracy in measuring MAP. The limits of agreement for systolic measurements were wider, and the trending ability compared to invasive measurements was outside the recommended limit. The ccNexFin is not reliable for CO measurements or trending ability in pregnant women undergoing caesarean delivery under spinal anesthesia.

## Data Availability

The datasets used and analyzed during the current study is available from the corresponding author on reasonable request.

## Abbreviations

CO: Cardiac output
BP: Blood pressure
HR: Heart rate
SAP: Systolic arterial pressure
MAP: Mean arterial pressure
IBI: Inter-beat interval
